# Neural and physiological relations observed in musical beat and meter processing

**DOI:** 10.1002/brb3.1836

**Published:** 2020-09-12

**Authors:** T. Christina Zhao, Patricia K. Kuhl

**Affiliations:** ^1^ Institute for Learning and Brain Sciences University of Washington Seattle WA USA

**Keywords:** beat and meter, heart rate variability, music processing, neural entrainment, physiology, rhythm

## Abstract

**Introduction:**

Music is ubiquitous and powerful in the world's cultures. Music listening involves abundant information processing (e.g., pitch, rhythm) in the central nervous system and can also induce changes in the physiology, such as heart rate and perspiration. Yet, previous studies tended to examine music information processing in the brain separately from physiological changes. In the current study, we focused on the temporal structure of music (i.e., beat and meter) and examined the physiology, neural processing, and, most importantly, the relation between the two areas.

**Methods:**

Simultaneous MEG and ECG data were collected from a group of adults (*N* = 15) while they passively listened to duple and triple rhythmic patterns. To characterize physiology, we measured heart rate variability (HRV), indexing the parasympathetic nervous system function (PSNS). To characterize neural processing of beat and meter, we examined the neural entertainment and calculated the beat‐to‐meter ratio to index the relation between beat‐level and meter‐level entrainment. Specifically, the current study investigated three related questions: (a) whether listening to musical rhythms affects HRV; (b) whether the neural beat‐to‐meter ratio differed between metrical conditions, and (c) whether neural beat‐to‐meter ratio is related to HRV.

**Results:**

Results suggest that while at the group level, both HRV and neural processing are highly similar across metrical conditions, at the individual level, neural beat‐to‐meter ratio significantly predicts HRV, establishing a neural–physiological link.

**Conclusion:**

This observed link is discussed under the theoretical “neurovisceral integration model,” and it provides important new perspectives in music cognition and auditory neuroscience research.

## Graphical abstract

The current study reported a neural–physiological link that has not been previously discussed. Specifically, neural processing of music rhythm and the activity of parasympathetic nervous system (PSNS) are significantly related across individuals. These results shed light a new research direction in music cognition.

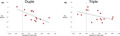

## 1 INTRODUCTION

Music is one of the most ubiquitously communicative sounds across the world's cultures. Listening to music is a powerful experience that engages the whole body. It not only requires neural processing of abundant musical information, such as the melody line, the rhythm, the instrument, and the genre, but it also induces responses in the physiology of the listeners, such as changes in heart rate and perspiration. So far, the physiological responses have been almost solely studied in the context of music‐evoked emotion, with the common approach to measure the physiology of the listeners while they listen to musical excerpts that are meant to elicit strong emotions. However, while it is known that physiological can also be affected by other factors, such as cognitive factors (Shaffer, McCraty, & Zerr, 2014), the relation between physiology and music information processing in the brain remains largely understudied.

The physiological responses related to music‐evoked emotion are robust and have been documented repeatedly (e.g., (Koelsch & Jäncke, 2015)). For example, when listening to happy, sad, and fearful music, participants were observed to show differential physiological response patterns, such as in blood pressure, heart rate, skin conductance, and respiration (Krumhansl, 1997). In another study, participants were assigned to either listen to relaxing music or silence after a stressful task. Participants who listened to relaxing music exhibited significantly more reduced cortisol level than the silent control group (Khalfa, Bella, Roy, Peretz, & Lupien, 2003). When participants experience a “chill” response (i.e., an intense emotional response) to music, it was observed that they demonstrated enhanced skin conductance and heart rate (Grewe, Kopiez, & Altenmüüller, 2009).

However, most of the measurements in these studies target the sympathetic nervous system (SNS) (e.g., heart rate, skin conductance, cortisol level). Parasympathetic nervous system (PSNS), another integral component of the autonomic nervous system, has largely not been a focus of music research (Wehrwein, Orer, & Barman, 2016). While the SNS is generally considered associated with arousal to prepare one for action, PSNS is thought of as bringing the system back to homeostasis (i.e., a balanced state) (Francis & Oliver, 2018). PSNS function is indexed by the measurement of heart rate variability (HRV), that is, changes in the variability of the time intervals between successive heartbeats (Berntson et al., 1997; Laborde, Mosley, & Thayer, 2017). Specifically, the higher the HRV, the stronger the influence PSNS has on the autonomic nervous system; moreover, very low resting HRV has been found to be associated with increased risk of disease (Thayer, Yamamoto, & Brosschot, 2010). A handful of studies have examined HRV during music listening, for example, HRV was found to be lower when listening to excitative music in comparison to sedative music (Iwanaga, Kobayashi, & Kawasaki, 2005) and the effect from excitative music may have been induced mainly by the change in tempo (Kim, Strohbach, & Wedell, 2018).

PSNS is an important topic to study in music perception as it serves as a possible bridge between music processing in the central nervous system and peripheral physiology. Multiple theories have been proposed (see a review by (Shaffer et al., 2014)) that connect HRV with important higher‐level functions, for example, social/emotional function and development (“Polyvagal theory”)(Porges, 2007), neural and cognitive functions (“neurovisceral integration model”) (Thayer, Åhs, Fredrikson, Sollers, & Wager, 2012; Thayer, Hansen, Saus‐Rose, & Johnsen, 2009), as well as self‐regulation (McCraty & Childre, 2010). Specifically, the “neurovisceral integration model” emphasizes a neural–physiological relation, mapping out anatomical pathways that connect the cerebral cortex to the heart (Thayer et al., 2012).

The current study aims to bridge the gap in the literature and examines the relation between central neural processing of music and peripheral physiological activities. Specifically, we focus on the HRV given the importance of PSNS and theoretical frameworks relating HRV to higher‐level neural processes. At the same time, most studies concerning physiology in the music emotion literature have used naturalistic music excerpts to elicit different emotions. While this is a more ecologically valid approach, it precludes us from understanding precisely which components of the music are connected to physiology and therefore effective for inducing changes. Therefore, in this study, we focus on a specific component of music, namely the temporal structure (i.e., beat and meter).

The temporal structure hierarchically organizes rhythmic information in music. The beat‐level temporal structure provides the sense of regular pulses that are perceived to occur at equal time intervals (i.e., isochronous); and the meter‐level temporal structure involves further organizing beats into groups or units through accenting some beats, for example, duple meter as marching rhythm (strong‐weak‐strong‐weak) and triple meter as waltz rhythm (strong‐weak‐weak‐strong‐weak‐weak). Beat and meter processing is a crucial skill as it has been shown to be associated with higher‐level cognitive skills, such as attention (Khalil, Minces, McLoughlin, & Chiba, 2013), syntactic skills (Gordon et al., 2015), and reading (Carr, White‐Schwoch, Tierney, Strait, & Kraus, 2014).

Various neuroscientific approaches have been used to understand how the brain processes beat and meter in music. To track beat, it has been shown that motor regions (e.g., basal ganglia, supplementary motor areas, premotor cortex), in addition to auditory regions, are serving crucial roles (Geiser, Notter, & Gabrieli, 2012; Grahn & Brett, 2007; Grahn & McAuley, 2009). Using MEG, additional studies have demonstrated brain activity in sensorimotor areas coupled with beat‐level frequency as well as beta‐band oscillatory patterns (highly associated with sensorimotor systems) that are related to processing of isochronous beats (Fujioka, Trainor, Large, & Ross, 2009, 2012; Morillon & Baillet, 2017). A recently proposed model suggests that a large subcortical–cortical network that incorporates the frontal cortex, basal ganglia, and cerebellum in addition to the auditory system is in play when processing beat‐level temporal structure (Schwartze & Kotz, 2013).

For meter‐level temporal structure processing, research has demonstrated that higher‐level processes play an important role and that this is reflected in neural responses. ERP studies have demonstrated that listeners’ cortical responses to identical sounds differ when the sounds were at metrically stronger locations in musical passages vs. weaker locations (Fitzroy & Sanders, 2015) and disruption to metrically strong locations elicit stronger neural responses (Brochard, Abecasis, Potter, Ragot, & Drake, 2003). Similarly, participants’ beta‐band oscillatory activity in response to isochronous tones was measured after they have been primed to hear the streams in metrical patterns (e.g., strong‐weak‐strong‐weak); beta‐band activities were observed to decrease more after perceptually stronger (or accented) beats than weaker beats even though the sounds were identical (Fujioka, Ross, & Trainor, 2015). In addition, neural detection of meter violation also differed based on the type of metrical structure (e.g., duple vs. triple), possibly due to different amounts of exposure (i.e., duple meter is more prevalent), although the exact nature of the difference remains unclear (Abecasis, Brochard, Granot, & Drake, 2005; Fujioka, Zendel, & Ross, 2010; Zhao, Lam, Sohi, & Kuhl, 2017).

In recent years, a new method has emerged to further study beat and meter processing, namely neural entrainment to beat and meter through frequency tagging (Nozaradan, 2014). That is, neural activity *entrains*, or modulates in a periodic manner, following the periodic beat and meter in the sound stream. Peaks in the power spectrum of the neural signal, at the frequencies that corresponds to periodicity related to beat and meter, can therefore be observed. In the original study, musicians listened to isochronous beats while their neural activities were measured by EEG. They were instructed to imagine the isochronous beats in different metrical structures (i.e., duple or triple). In the power spectrum of their recorded EEG, peaks at these imagined meter‐level frequencies were observed along with the peak at the beat‐level frequency, demonstrating that meter processing involves mental grouping and the organization of beats (Nozaradan, Peretz, Missal, & Mouraux, 2011). Using this method, the authors further observed that the neural entrainment to beat and meter‐related frequencies are selectively amplified compared to unrelated frequencies in complex rhythms, providing more evidence of the endogenous aspect of beat and meter processing (Nozaradan, Peretz, & Mouraux, 2012). Moreover, using ecologically valid music, it was demonstrated that entrainment to meter, not beat, can be disrupted with conflicting cues, further showing that meter processing may involve higher‐level processes (Tierney & Kraus, 2015). Most recently, Li and colleagues examined neural entrainment using simultaneous EEG‐fMRI and observed networks that are distinct to beat vs. meter entrainment (Li et al., 2019).

**Figure 1:**
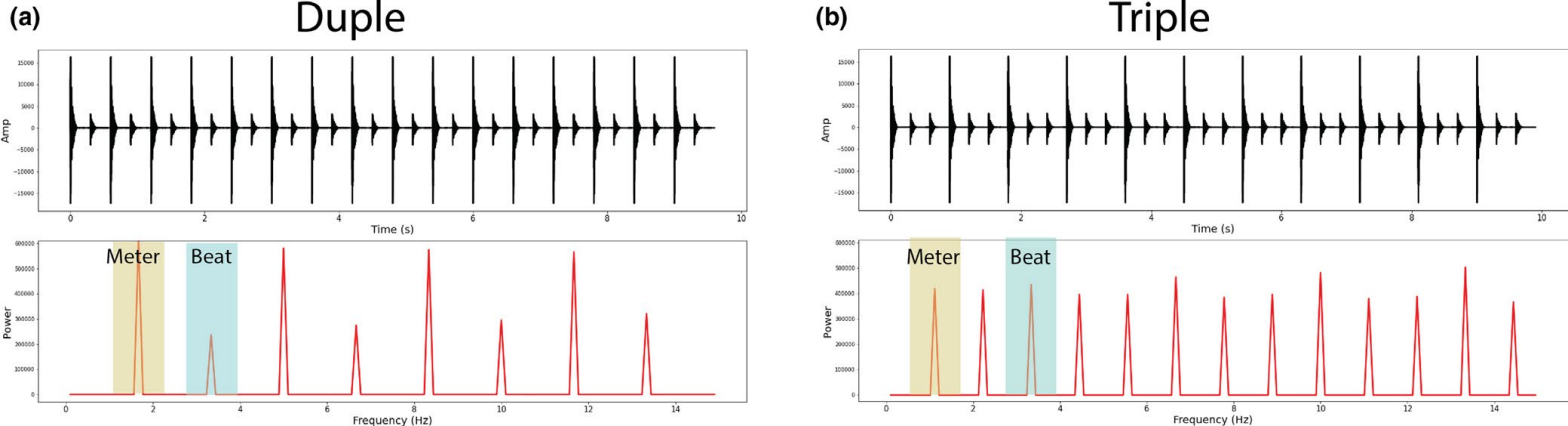
Stimulus waveforms (top panel) and power spectra (bottom panel) for (a) duple and (b) triple condition. In the power spectra, peaks at beat‐level frequencies are shaded in blue while peaks in meter‐level frequencies are shaded in yellow

The current study adapted the neural entrainment method to examine the neural processing of music beat and meter. Adult participants passively listened to 5‐min blocks of duple and triple meter rhythm while their neural activities were measured by magnetoencephalography (MEG) (Figure 1a). We also measured their simultaneous cardiac activities using ECG during these blocks. In addition, resting ECG was also measured for 5 min prior to the sound presentation. Following the neural tagging method, we first calculated the power spectrum of the MEG signal and focused on the power values at the beat‐level frequency and the meter‐level frequency. Different from previous studies, we further calculated the ratio between the power values for beat‐level and meter‐level frequencies (i.e., beat‐to‐meter ratio = beat‐level power value/ meter‐level power value) for each individual. This ratio indexes the relation between beat and meter processing, that is, the higher the ratio, the more neural entrainment tags the beat while the lower the ratio, the more neural entrainment tags the meter. For the ECG data, we extracted heart rate variability (HRV) measures that can index PSNS function. Our main question concerns the relation between neural processing and physiology; in addition, we are interested in whether there are differences between duple and triple meter conditions given that previous studies have reported processing differences between the two meters (Abecasis et al., 2005; Fujioka et al., 2010; Zhao et al., 2017).

We addressed three specific questions: (a) Whether HRV differed across resting state, duple meter, and triple meter conditions. We hypothesized that HRV will be higher during resting state than during sound presentation. We did not have specific hypotheses regarding differences between triple and duple meter conditions. (b) We examined the beat‐to‐meter ratio between duple and triple meter conditions, and we anticipated differences between the two conditions, given the difference in difficulty for error detection between these two conditions (Zhao et al., 2017). (c) Most importantly, we conducted regression analyses to examine relations between the neural beat‐to‐meter ratio and HRV at the individual level.

## 2 METHOD

### 2.1 Participants

16 individuals were recruited to participate in this study at the University of Washington. One individual opted to discontinue after consent. The age of the 15 participants (7 males) ranged from 23 to 32 years old (mean age = 26.87 years [*SD* = 2.77]). None of the participants reported any speech, language, or hearing difficulties. Three participants reported growing up with another language aside from English in the household. Others were native, monolingual speakers of American English. Self‐reported formal music training (i.e., private lessons) ranged from 0–17 years (mean = 6.48 years [*SD* = 6.16]). All procedures were approved by the University of Washington Institution Review Board, and all participants were compensated monetarily for their participation.

### 2.2 Stimuli

Two stimuli (~10s long each) were generated that varied in metrical structure (i.e., duple vs. triple meter). The metrical structures were established by playing strong and weak complex tones in specific sequences in a stream (Figure 1 Top Row shows stimulus waveforms). In both stimuli, the inter‐onset interval was 300ms. The complex tone (Duration: 100ms, Sampling frequency: 44.1kHz) was synthesized to have a fundamental frequency of 220 Hz (A3) and a “grand piano” timbre along with a “woodblock” sound in the Overture software (Version 4, Sonic Scores). This complex tone (weak tone) was amplified by 10dB in Audacity software (Version 2.0, Sound Forge) to create the strong tone. Such stimuli have been widely used in the literature (Hannon & Trehub, 2005b; Winkler, Haden, Ladinig, Sziller, & Honing, 2009; Zhao et al., 2017).

The spectra of the stimuli, calculated through Fourier transform, can be visualized in Figure 1 Bottom Row. In both stimuli, beat‐level rhythm energy has a peak at 3.33 Hz (corresponds to the ISI of 300ms), while meter‐level rhythm energy has a peak at 1.67 Hz (corresponds to inter‐strong‐beat intervals of 600ms) for duple meter and 1.11 Hz (corresponds to inter‐strong‐beat intervals of 900ms) for triple meter.

### 2.3 Experimental design and procedure

During an experimental session, participants were first consented and then completed a brief language and music background questionnaire. Simultaneous MEG and ECG recordings were completed inside a magnetically shielded room (MSR)(IMEDCO America Ltd, IN). For MEG, an Elekta Neuromag system was used with 204 planar gradiometers and 102 magnetometers. In preparation, five head position indicator (HPI) coils were attached to each participant's head. Head positions under the MEG dewar were collected from these sensors at the beginning of each block. Then, three landmarks (left preauricular, right preauricular, and nasion) and the HPI coils were digitized along with 100 additional points along the head surface with an electromagnetic 3D digitizer (Fastrak®, Polhemus, Vermont, U.S.A). In addition, a pair of electrocardiography sensors (ECG) were placed on the front and backside of the participants’ left shoulder to record cardiac activity and 2 pairs of electro‐oculogram (EOG) sensors were placed (one pair placed above and below left eye and one pair lateral to each eye) to measure eye blinks and saccades.

The stimuli were processed such that the RMS values were referenced to 0.01, and it was further resampled to 24,414 Hz for the TDT (Tucker‐Davis Technologies Inc., FL). The normalization process ensured that the overall intensity level of the stimuli was equal; however, the power relation for the beat and meter frequencies are different as can be seen in Figure 1b. Then, the stimuli were delivered from a TDT RP 2.7 device, controlled by custom python software on a HP workstation to a speaker with a flat frequency response at a comfortable listening level of 65 dB SPL, measured under the MEG dewar. The stimuli were presented in blocks (~6 min each block). In each block, the same trial (~10s long) was repeated 30 times with ~2s breaks between repetitions. The order of the blocks was randomized across individuals.

The participants listened passively and watched silent videos during recording. And all recordings were completed using a 1,000 Hz sampling rate. A 5‐min long empty room recording was done prior to each participant's session. In addition, a 5‐min resting‐state ECG recording was also done for each participant prior to stimulus presentation.

### 2.4 Data processing

HRV analysis was done using in‐house MATLAB software for processing ECG data. QRS complexes (peaks indexing heartbeats) were first identified through algorithms and were then manually edited to ensure that all peaks were correctly labeled. The inter‐beat intervals (IBIs) were subsequently extracted and used to calculate the RMSSD for each condition, the outcome measure for HRV. The RMSSD is defined as the square root of the mean of the sum of the squares of differences between adjacent inter‐beat intervals (IBIs). The measurements and analysis have been well established and standardized in the field of HRV research (Laborde et al., 2017).

All MEG processing and analyses were done using the MNE‐python software (Gramfort et al., 2013). MEG data were first preprocessed using the temporally extended spatial signal separation (tSSS) method (Taulu & Hari, 2009; Taulu & Kajola, 2005) and head movement compensation to suppress sensor noise and magnetic interference originating from outside of the MEG dewar. MEG data were low‐pass filtered at 40  Hz and high‐pass filtered at 0.1 Hz. The artifacts from heart beats as well as eye movements were further suppressed using the signal‐space projection method (SSP)(Uusitalo & Ilmoniemi, 1997) as implemented in MNE‐python. Epochs (−1 to 11s) were extracted and averaged to generate the evoked responses to the stimuli. Subsequently, the power spectral density (PSD) of the evoked response for each sensor was calculated using the Welch's method (Welch, 1967) in MNE‐python, and then, the PSDs were averaged across sensors with 0.05 Hz resolution. Individual‐ and group‐level PSD for Duple and Triple conditions can be visualized in Figure 3a and Figure 3b. Peak values between 3.2 and 3.5 Hz were extracted for beat‐level energy for both conditions. For meter‐level energy, peak values between 1.6 and 1.9 were extracted for the duple condition and peak values between 1.0 and 1.4 were extracted for the triple condition. Mean frequency values at which beat‐level energy occurs are 3.35 Hz (*SD* = 0.01) for duple condition and 3.35 Hz (*SD* = 0.00) for the triple condition. Mean frequency values at which meter‐level energy occurs are 1.65 Hz (*SD* = 0.00) for duple condition and 1.11 Hz (*SD* = 0.02) for the triple condition. The mean power for the beat‐level energy was 3.94 × 10^–25^ Tesla^2^ (*SD* = 1.56) for the duple condition and 3.82 × 10^–25^ Tesla^2^ (*SD* = 1.77) for the triple condition. The mean power for the meter‐level energy was 2.05 × 10^–25^ Tesla^2^ (*SD* = 1.61) for the duple condition and 1.94 × 10^–25^ Tesla^2^ (*SD* = 1.16) for the triple condition. The beat‐to‐meter ratio was calculated by dividing the beat‐level peak energy by the meter‐level peak energy for each individual.

Power spectral density (PSD) was also calculated at the source level for each individual for secondary explorative analyses. Forward modeling used the boundary element method (BEM)‐isolated skull approach with inner skull surface extracted from the averaged adult brain (Fischl, Sereno, Tootell, & Dale, 1999). Both the source space and the BEM surface were then aligned and scaled to optimally fit each subject's head shape revealed by head digitization points. Inverse source modeling was performed on the sensor power spectral density, using the dynamic statistic parametric mapping method (dSPM) without dipole orientation constraints and with data from both gradiometers and magnetometers (Dale et al., 2000). The source activities were normalized to the noise covariance computed from the corresponding empty room recording, which underwent the same preprocessing steps except for the movement compensation. This procedure resulted in statistically normalized scores for 3 dipole components at each source location for each frequency (i.e., dipole strengths in 3 orthogonal directions). Lastly, the magnitude was calculated across the 3 dipole components at each source location and was taken as the power spectral density for that source location. Left hemisphere PSD was calculated by averaging across all source locations in the left hemisphere and similarly, and right hemisphere PSD was calculated by averaging across all source locations in the right hemisphere. The beat‐to‐meter ratios were extracted and calculated separately from the left and right hemisphere PSD, using the same method as for the sensor‐level analysis.

## 3 RESULTS

**Figure 2:**
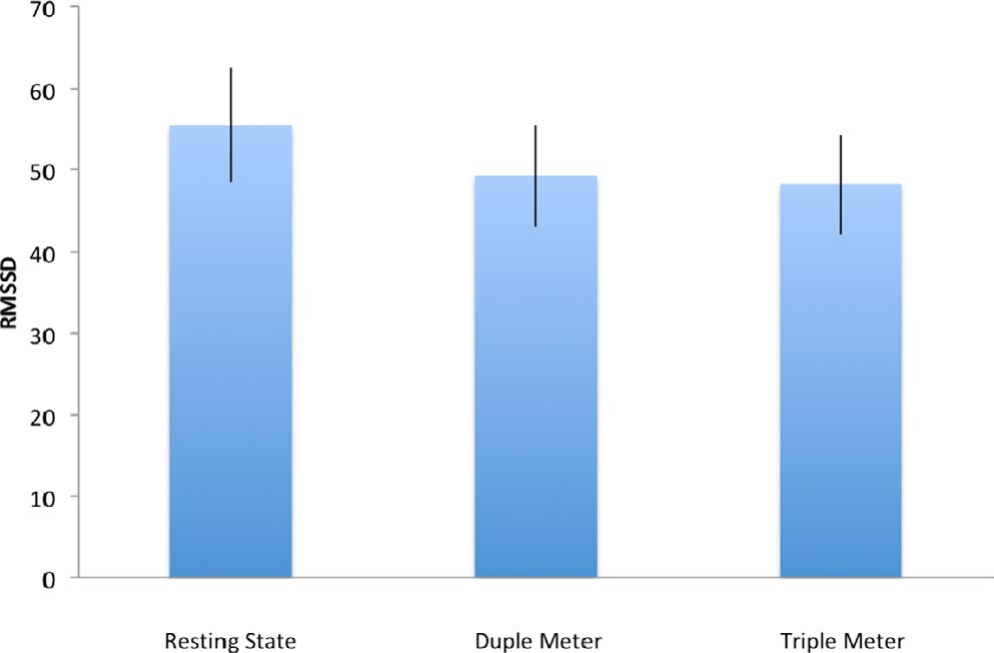
Heart rate variability across resting state, duple meter, and triple meter conditions

The data reported in this study are openly available in Open Science Framework (Zhao, 2019). To address question 1, we conducted a repeated measures analyses of variance (ANOVA) of HRV across the resting condition, duple meter condition, and triple meter condition (SPSS Version 19). Results (Figure 2) show a significant main effect of conditions (*F* (2, 28) = 4.760, *p* = .035 with Greenhouse‐Geiser correction), η^2^ = 0.234). *Post hoc* comparisons between conditions with Bonferroni correction suggest that the effect was mainly contributed to by a significant difference (*p* = .039) between resting‐state condition (mean = 55.18, 95% CI [39.99, 70.34]) and duple meter condition (mean = 48.9, 95% CI [35.66, 62.14]. No significant differences were observed between duple and triple meter condition (Triple mean = 48.95, 95% CI [35.95, 61.94], *p* = 1.00), or between resting state and triple meter condition (*p* = .18).

**Figure 3:**
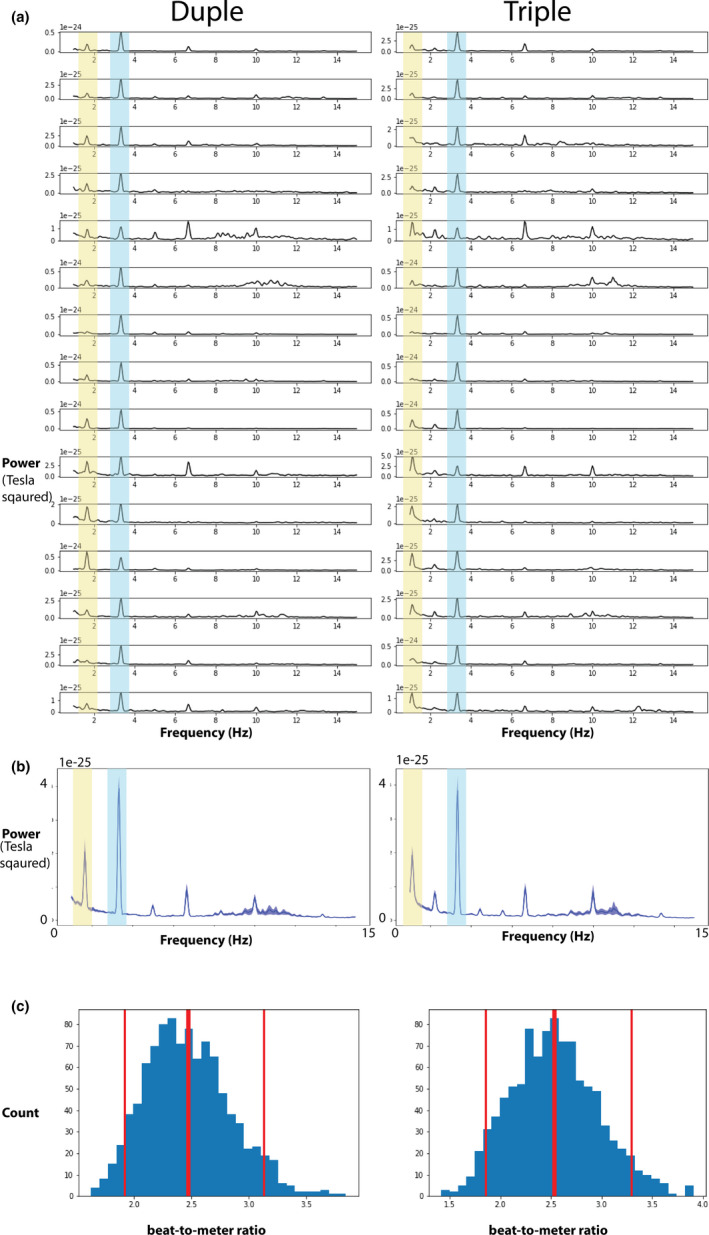
(a) Individual power spectrum density for duple (left) and triple (right) conditions. Blue shaded peak corresponds to beat‐level rhythm, and yellow shaded peak corresponds to meter‐level rhythm. (b) Group average power spectrum density for duple (left) and triple (right) conditions. (c) Bootstrap distributions of beat‐to‐meter ratios over 1,000 simulations for duple (left) and triple (right) conditions. Mean of distribution (center red vertical line) and 95% confidence interval (left and right red vertical lines) are marked shown

To address question 2, we estimated the mean of sensor‐level beat‐to‐meter ratios using the boostrap method with 1,000 repetitions (Efron & Tibshirani, 1994). The bootstrap mean of beat‐to‐meter ratio is 2.48 with a 95% confidence interval between [1.91, 3.15] in the duple condition (Figure 3c left column). Similarly, the boostrap mean of beat‐to‐meter ratio for the triple condition is 2.52 with 95% confidence interval between [1.79 3.31] (Figure 3c right column). Further, we explored whether there was a hemisphere difference in the beat‐to‐meter ratio for each condition. Related samples Wilcoxon signed rank tests (SPSS Version 19) were used to compare left and right hemisphere beat‐to‐meter ratio as the ratios did not follow a normal distribution as shown by the Kolmogorov–Smirnov test. For both conditions, no hemisphere differences were observed (*p* = .75 for duple condition and *p* = .91 for triple condition). The results suggest that despite the different ratios observed in the sound stimuli between beat and meter energy (duple condition: beat‐to‐meter ratio in stimulus = 0.39, triple condition: beat‐to‐meter ratio in stimulus = 1.04), in adults, the brain tracks the beat‐level rhythm about 2.5 times stronger than the brain tracks the meter‐level rhythm at the group level.

**Figure 4:**
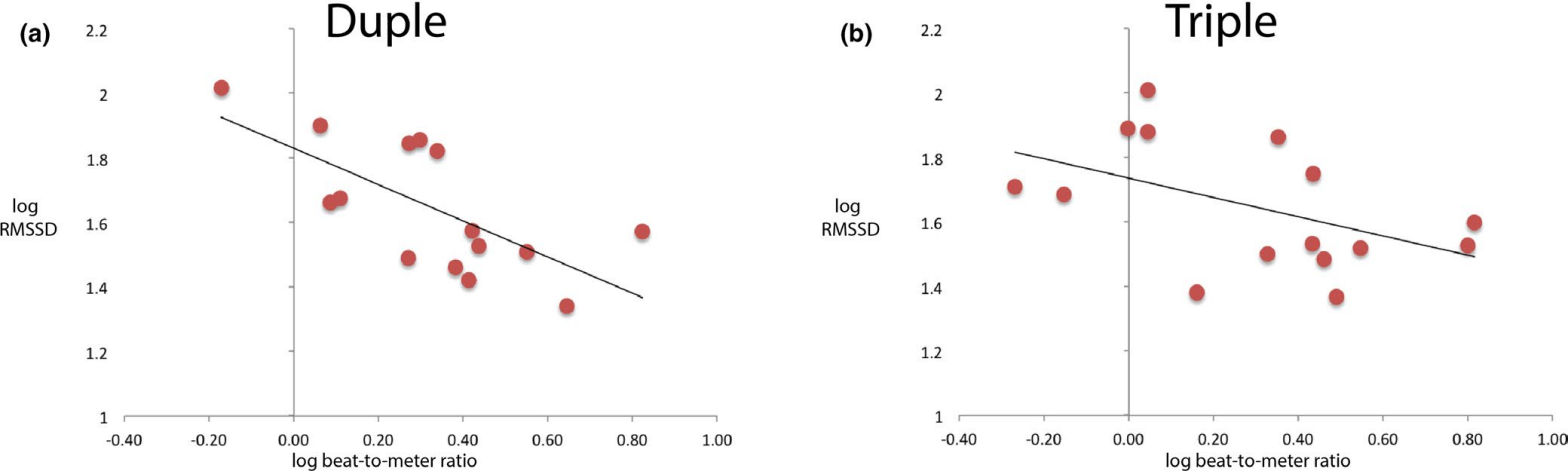
Scatter plots between log‐transformed beat‐to‐meter ratio and log‐transformed RMSSD for A) duple and B) triple conditions. The best fitting regressions lines are shown on the graph

To address question 3, we conducted regression analyses between beat‐to‐meter ratios (i.e., neural processing measure) and RMSSD (i.e., physiological HRV measure). Both beat‐to‐meter ratios and RMSSD were first log‐transformed to ensure normal distribution. Regression analyses suggested a significant linear relation between beat‐to‐meter ratio and RMSSD in the duple condition (Pearson r = 0.694, R‐square = 0.481, *p* = .004, slope = −0.56, intercept = 1.83) (Figure 4a) and a marginally significant linear relation between the two measures in the triple condition (Pearson r = 0.485, R‐square = 0.23, *p* = .067, slope = −0.29, intercept = 1.74) (Figure 4b).

We conducted a few additional explorative analyses. First, we explored the relations between resting‐state RMSSD with sensor‐level beat‐to‐meter ratios. Results showed that resting‐state HRV is correlated to beat‐to‐meter ratio only in the duple condition (Pearson r = −0.524, *p* = .045), not in the triple condition (Pearson r = −0.239, *p* = .391). Second, we explored relations between RMSSD with left and right hemisphere beat‐to‐meter ratios for potential hemispheric differences. Correlational analyses suggest that in both conditions, significant correlations were only observed between right hemisphere beat‐to‐meter ratio and RMSSD (duple condition: Pearson r = −0.55, *p* = .033; triple condition: Pearson r = −0.70, *p* = .003). There were no significant relations observed between left hemisphere beat‐to‐meter ratio and RMSSD (Duple condition: *p* = .276; Triple condition: *p* = .189).

Finally, we explored whether music training background is associated with either beat‐to‐meter ratio or RMSSD measures. Years of private music lessons were taken as a measure for music training background, similar to previous studies (Zhao & Kuhl, 2015). No significant correlations were observed between music training and either measures in either condition (all *p* > .5).

## 4 DISCUSSION

The current study examined three interrelated research questions that aimed to shed light on the connection between neural processing of music and physiological responses to music. Specifically, we focused on beat and meter processing and measure the neural entrainment to beat and meter in duple and triple metrical structures. We calculated the ratio between the power values at beat‐level and meter‐level frequencies (i.e., beat‐to‐meter ratio) to index the relation between beat and meter‐level neural entrainment in individuals. We also measured the heart rate variability (HRV), a key index of the PSNS function prior to and during sound presentation. Our first question tested whether HRV differed between the three conditions (i.e., resting, duple meter, and triple meter). Results suggest that, as expected, HRV decreased during sound presentation, indexing decreased PSNS function as the brain activates to process incoming musical information. However, at the group level, there was no difference between duple and triple meter condition. Similarly, for our second question, despite the difference between the spectra of stimuli of duple and triple condition, as well as previous research suggesting differential processing for duple and triple meters, the results showed nearly identical beat‐to‐meter ratios between duple and triple meters at the group level, demonstrating very similar relations between beat and meter processing across conditions. Most interestingly and importantly, at the individual level, the beat‐to‐meter ratio characterizing neural processing of musical beat in relation to meter, significantly predicts HRV values, indexing PSNS function, supporting a link between neural processing and physiological response to music. More specifically, the more the brain tracks the meter‐level rhythm (i.e., lower beat‐to‐meter ratio), the higher the HRV(i.e., higher PSNS function, which is generally interpreted as a closer to homeostasis, less‐stressed state).

To date, physiological responses have mostly been studied in the context of music emotion, with physiology being an integral aspect of emotion measurement along with subject rating and behavioral responses (Swaminathan & Schellenberg, 2015). However, little is known how physiology is related to music processing. At the group level, we observed a reduction in HRV from resting state to music processing blocks but there was no difference between duple and triple conditions. The lack of difference between duple and triple meter in HRV can be explained from two perspectives: (a) The stimuli are highly reduced and are not meant to elicit any emotional responses. Therefore, processing duple vs. meter alone does not affect physiology. (b) The tempo of the two conditions are the same (i.e., inter‐beat‐intervals are both 300 ms). Given that previous research demonstrated the effect of tempo on physiological responses (Gomez & Danuser, 2007; Kim et al., 2018), future studies may further test whether tempo is an crucial component of music‐evoked emotion by examining whether tempo alone affects both HRV and subject rating of emotion, even with highly reduced musical beats.

The highly similar group‐level beat‐to‐meter ratios between conditions are surprising and interesting. The ratio is the same at around 2.5 for both conditions despite the fact the ratios were very different in the actual sound stimuli and that previous studies have demonstrated differences between duple and triple meter processing (Abecasis et al., 2005; Zhao et al., 2017). This result provides important new information regarding beat and meter processing that has not been previously examined, that is, the relation between beat and meter processing by calculating the beat‐to‐meter ratio. Previous studies have largely examined beat and meter processing separately. For example, the most common analyses consider beat‐level processing as a group and meter‐level processing as a group (Li et al., 2019; Nozaradan et al., 2011). Here, we show that while there may be differences for beat and meter processing, there may be a neural transformatory mechanism that organizes and represents meter‐level and beat‐level information in a set proportion to each other. Although not reported explicitly in previous papers, similar ratios can be visualized in the figures when participants merely imagined the metrical structures (Nozaradan et al., 2011). Future replication studies are warranted to further validate this idea by adding a wider range of rhythmic stimuli with various beat and meter frequencies and test a larger sample of subjects. On the other hand, our current finding is different from previous studies showing processing differences between duple and triple meters (Abecasis et al., 2005; Zhao et al., 2017). Previous studies involve maintaining the metrical temporal structure and detecting violations while the current study only involves maintaining the structure. It is possible that only during the more demanding tasks are differences between conditions observed. Future studies will need to systematically examine the effect of task demand as well as the utility of attention (passive vs. active) on beat and meter processing.

Most importantly, our finding that the beat‐to‐meter ratio at the individual level is highly predictive of HRV, an indicator of PSNS function, is novel. This suggests a link between neural processing of music information and physiology across individuals. More specifically, the higher the HRV (more PSNS function), the lower the beat‐to‐meter ratio, that is, the neural processing focuses more on the higher‐level meter processing.

Our finding should be distinguished from neuroimaging research that focuses on the neural mechanisms underlying music emotion (Koelsch, 2014), in which highly evocative music is used to elicit strong emotion. Here, we use highly reduced stimuli to study music information processing (i.e., beat and meter) and observed a robust neural–physiological connection. This result is consistent with the “neurovisceral integration model” (Thayer et al., 2009), in which a neural network that is responsible for higher‐level information processing (cognitive, social, emotional), including the prefrontal cortex, cingulate cortex, and insula, can affect and regulate cardiac activities through a series of subcortical structures in a top‐down manner. Given the correlational nature of this relation, the causal relation between music processing and physiological response remains elusive. Our *post hoc* analyses may suggest that an individual's general state (higher vs. lower HRV) is related to how their brain tends to process music beat and meter, given that the resting‐state HRV prior to stimulus blocks is correlated to beat‐to‐meter ratio, but only in the duple condition. Further research can take the approach to manipulate resting‐state HRV (e.g., given participants a stressful task) and then measure neural processing of a wider range of rhythmic stimuli to elucidate the causal relations. Future research should also incorporate subjective and behavioral evaluation of emotional response to map out the relations between physiology, music processing, and music emotion.

There were several interesting findings from the secondary analyses as well. While there were no hemisphere differences with regard to the beat‐to‐meter ratios for either condition at the group level, significant correlations were observed only between right hemisphere beat‐to‐meter ratios and HRV. These results are in line with (a) the idea that music rhythm processing relies on distributed neural networks in both hemispheres (Doelling & Poeppel, 2015; Fujioka et al., 2010), but that (b) the right hemisphere HRV correlation may suggest that the top‐down connection from the brain to the heart may rely more on contralateral pathways originating from the right hemisphere.

It is natural to ask whether such connections can be modulated by experience as well as development. Our *post hoc* analysis suggests that years of music training are not related to either the beat‐to‐meter ratio or HRV, which points to a more fundamental mechanism. However, many studies have found music training‐related effects in beat and meter processing (Doelling & Poeppel, 2015; Zhao et al., 2017). Future studies will need to disentangle the differences between the paradigms and measurement methods and identify processes that are basic to beat and meter processing as opposed to ones that can be modulated by experience. Another approach in future research would be to examine the effects of experience and development by studying developmental populations. For example, previous studies have demonstrated changes in beat and meter processing in infancy that are related to the musical environment that infants experienced. These changes can now be simulated by a learning model (Hannon & Trehub, 2005a, 2005b; Tichko & Large, 2019).

## 5 CONCLUSION

The current study reported on an important link between neural processing of music beat and meter information and the physiological response to it across individuals. This neural–physiological link has not previously been reported, and it sheds light on the research direction that examines the interconnection between music cognition and emotion.

## CONFLICT OF INTEREST

The authors report no conflict of interest.

## AUTHOR CONTRIBUTION

The first author designed the study, collected, and analyzed the data and contributed to manuscript preparation. The second author contributed to the manuscript preparation.

### Peer Review

The peer review history for this article is available at https://publons.com/publon/10.1002/brb3.1836.

## Data Availability

The data reported in this study are publicly available in Open Science Framework (Zhao, 2019).
